# A pharmacist health coaching trial evaluating behavioural changes in participants with poorly controlled hypertension

**DOI:** 10.1186/s12875-021-01385-0

**Published:** 2021-02-14

**Authors:** Harjit K. Singh, Gerard A. Kennedy, Ieva Stupans

**Affiliations:** 1 Discipline of Pharmacy, The School of Health and Biomedical Sciences, RMIT University VIC, Bundoora, VIC 3083 Australia; 2 School of Health and Life Sciences, Federation University, University Drive, Mount Helen, Ballarat, Melbourne, Australia; 3 Institute for Breathing and Sleep, Austin Health, 145 Studley Road, Heidelberg, Melbourne, Australia

**Keywords:** Health coaching, Community pharmacist, Hypertension, Behaviour change, Stages of change, Medication adherence

## Abstract

**Background:**

To investigate whether pharmacist health coaching improves progression through the stages of change (SOC) for three modifiable health behaviours; diet, exercise, and medication management in participants with poorly controlled hypertension.

**Methods:**

In this four-month controlled group study two community-based pharmacists provided three health coaching sessions to 20 participants with poorly controlled hypertension at monthly intervals. Changes in participants’ stages of change with respect to the modifiable health behaviours; diet, exercise, and medication management were assessed. To confirm the behaviour change outcomes, SOC were also assessed in a control group over the same period.

**Results:**

Statistically significant changes in the modifiable health behaviours- medication management (*d* = 0.19; *p* = 0.03) and exercise *(d* = 0.85*; p* = 0.01) were apparent in participants who received health coaching and were evident through positive changes in the SOC charts. The participants in the control group did not experience significant changes with respect to the SOC. This was parallel to a decrease in mean systolic blood pressure from session one to session four by 7.53 mmHg (*p* < 0.05, *d =* − 0.42) in participants who received health coaching. Improvements to medication adherence was also apparent in these participants, evident from the mean scores for the Adherence to Refills and Medications Scale (ARMS), which decreased significantly from a mean of 15.60 to 13.05 (*p* < 0.05) from session one to four.

**Conclusions:**

Pharmacist health coaching produced promising health outcomes in participants with poorly controlled hypertension. Pharmacists were able to facilitate a positive behaviour change in participants. However, larger participant cohorts are needed to explore these findings further.

**Trial registration:**

Australia New Zealand Clinical Trials Registry ACTRN12618001839291. Date of registration 12/11/2018.

**Supplementary Information:**

The online version contains supplementary material available at 10.1186/s12875-021-01385-0.

## Background

Hypertension is one of the most important preventable risk factors contributing to premature cardiovascular morbidity and mortality in Australia [1–3]. Essential hypertension is defined as having persistent systolic and diastolic blood pressure (BP) of 140 and 90 mmHg or higher, respectively [4]. Although not curable, hypertension, under the guidance of a qualified health care professional, is a manageable lifelong condition. However, despite this, a considerable number of Australians diagnosed with hypertension have poorly controlled BP [3]. Lifestyle behaviours, poor medication management, lack of disease knowledge, and insufficient monitoring are contributors to chronic illnesses such as hypertension [5]. Consequently, the cost of treating these diseases is increasing dramatically [6]. Interventions that target behaviour change emphasise participant accountability and, consequently, lower costs and are imperative in reducing the burden on primary health care infrastructures [7]. However, to facilitate these changes, healthy lifestyle programs must address the barriers to chronic disease management as well as a person’s readiness to make a lifestyle change [8].

In Australia, the provision of care in the community is complicated because general practitioners (GPs) are pushed for time and there are low numbers in rural and remote areas [9]. Furthermore, GPs traditionally work business hours [9]. Considering community pharmacists are more easily accessed than other primary care providers as they are available after hours [9] and without the need for appointments, they are in an ideal position to assist participants in the monitoring and management of chronic health conditions by influencing and reinforcing appropriate lifestyle changes [3].

Pharmacist health coaching is a promising professional pharmacy service, helping individuals change health behaviours [10–12]. Health coaching is defined as a service that is provided to participants by health care professionals for disease management. It involves a collaborative participant-centred interaction between the participant and coach for the purpose of behavioural change through the process of goal setting and follow-up. Both the coach and participant are held accountable for the participants’ outcomes, though it is the role of the coach to provide expert information and facilitate the motivation of the participant in order to achieve their goal [13].

A series of publications from the United States (US) have shown that pharmacist health coaching has produced favourable outcomes in participants with chronic health conditions such as hypertension [13–17]. One study reported that although the reduction in systolic BP change was not statistically significant, 90% of participants were satisfied with the service and care that they received from the pharmacists [16]. In a second US pharmacist health coaching study evaluating the clinical and economic outcomes [17] results showed that in hypertensive participants who received health coaching adherence to medications increased 11% (*p* < 0.05) and BP improved significantly from 136.1/83.5 mmHg at baseline to 129.5/79.3 mmHg at follow-up (p < 0.05). These changes have correlated with a reduction in hypertension-related healthcare costs [17], and also indicate that pharmacist health coaching provides a way to assist participants with health education, medication adherence, prevention, and management of hypertension improving both clinical and non-clinical parameters.

The effectiveness of pharmacist health coaching interventions for participants with chronic health conditions has been assessed using clinical and non-clinical outcome measures, including validated medication adherence questionnaires, clinical targets, and cost-effectiveness [14, 16, 17]. However, previous studies have not investigated the health behaviours involved in improving these single algorithmic measures. By focusing on several behaviours such as; medication, adherence, management, diet, exercise, to assess a participant’s improvement in chronic illness, pharmacists will be able to more effectively assess readiness to change, leading to improved and targeted treatment [18].

The behaviour change process that occurs during health coaching can be described by several types of psychological models and theories. One of the most frequently referred to in the literature is the Transtheoretical Model of Change (TTM) [19]. The Transtheoretical Model of Change is also referred to as the stages of change (SOC) approach, as it involves five stages of change: (1) pre-contemplation; (2) contemplation; (3) preparation; (4) action; and (5) maintenance [19]. Although each stage can be defined separately, motivation and readiness to change are important components in the participants’ progress through the SOC cycle [20]. Movement from one stage to the next is not possible without fulfilling the previous stage [20]. Therefore, the behaviour change process, as part of this theory, is described as a dynamic cyclic process in which at any one time, an individual is in one of the stages, but may move forward to the next stage (progress) or backwards to the previous stage (relapse) [8]. Health coaches can utilise this assumption, identifying an individual’s position within the SOC to guide them to fulfil specific tasks involved in each stage thus promoting internal readiness and motivation to change [19].

Several studies involving health care professionals have used techniques such as counselling or coaching to apply the TTM. The approach has been shown to improve the management of behaviours related to alcohol abstinence, medication adherence, and dietary restraint. The TTM produces favourable health outcomes by encouraging participants to move forward through the SOC. [21, 22] Pharmacists currently use the SOC approach to assist participants with smoking cessation [23, 24], but as far as can be determined from the literature, they have not yet applied the model to other health behaviours.

In this paper, a controlled study coaching intervention was implemented in an Australian community pharmacy. The intervention was evaluated pre- and post- the four-month trial period. The progression through the SOC for three modifiable health behaviours (diet, exercise, and medication management) in addition to the reduction of systolic blood pressure and improvement of medication adherence was evaluated.

## Methods

### Study design

A controlled study design was used. Participants with poorly controlled essential hypertension received pharmacist health coaching once a month for 3 months, followed by an end of study visit. The primary outcome was a change in participant’s SOC for three modifiable health behaviours: diet, exercise, and medication management before and after the health coaching intervention. In addition, the study sought to evaluate participants’ systolic blood pressure and medication adherence over the same period. To substantiate the findings of the SOC, participants with poorly controlled hypertension in a control group who did not receive health coaching were also assessed for their SOC with respect to the three modifiable health behaviours.

The study was conducted in Melbourne, Australia, and was prospectively registered for inclusion in the Australia New Zealand Clinical Trials Registry ACTRN12618001839291 on November 12, 2018. The study received approval by the RMIT Human Research Ethics Committee (HREC project number: 21778) on December 21, 2018. Upon completion of the trial, intervention group participants received a $50 gift voucher.

### Participants and recruitment

Pharmacists from five community pharmacies in metropolitan Melbourne were approached to participate in the study. Given that the study involved regular site visits and monitoring, pharmacies that were easily accessible to the researchers were approached for the study. The main barriers to the participation of pharmacists in this research were that they were too busy and, lacked sufficient time and that there was no remuneration for their time.

From January 2019 to July 2019, pharmacists were asked to recruit 20 participants for the controlled trial. Inclusion criteria for participants were: (1) aged over 18 years; (2) diagnosed with essential hypertension (≥ 140/90 mmHg) by their GP; (3) taking at least one antihypertensive; (4) recognised as having poorly controlled essential hypertension by a pharmacist (determined using dispensing history records to assess compliance and participant/pharmacy BP records); and (5) understands the English language. Participants were excluded from the trial if they were a current smoker or had stopped smoking within the last 6 months.

A second group of 10 control participants with poorly controlled hypertension were also recruited. These participants did not receive health coaching and it was expected no changes would be apparent in their SOC charts between session one and session four.

### Pharmacist training

Pharmacists were trained on how to recognise and recruit eligible participants and how to health coach participants. The health coach training was provided by a registered pharmacist who was a member of the research team. Sessions were face-to-face and were conducted at a time and place convenient for the pharmacist. The training involved an interactive discussion between the pharmacist and a member of the research team. This included a mixture of didactic information about health coaching, the behaviour change process conceptualised within the transtheoretical model, as well as interactive periods during which pharmacists could ask questions and engage in role-plays. The study protocol and the process of the health coaching sessions was outlined to the pharmacists, this included instruction on how to tailor sessions to participants and how to motivate participants to progress through the SOC. A proforma for each health coaching session, which provided further guidance, was also given to the pharmacists.

### Pharmacist health coaching

Pharmacists provided monthly health coaching sessions to intervention group participants for 3 months. The expected duration of each health coaching session was 15–30 min, but this could be affected by factors such as the participant’s interest, pharmacist/participant time constraints, and general conversation. Depending on the availability of the participant, the first health coaching session occurred at approximately one-month post-enrolment in the study. Participants were educated about hypertension, associated complications, treatment options, and clinical targets, to improve their knowledge and attitudes about hypertension.

The three health coaching sessions employed the following format:

1. Record current blood pressure and compare it to the previous month.
2. Discuss diet, exercise, and medication adherence goals from the previous month.
3. Participant to set blood pressure goal for the following month.
4. Participant to set goals for diet, exercise, and medication adherence for the following month.

Note that discussions about a previous month were not applicable at the first health coaching session.

Session four took place 1 month after the completion of all health coaching sessions, at which the assessments from session one were repeated. An additional assessment took place at 3 months post-study completion and participants were contacted for a telephone interview, with the intent to follow-up on their progress and to investigate whether participants were making use of the skills learned during health coaching sessions at the pharmacy.

### Outcome measures

The primary outcome measure was progression through the SOC for three modifiable health behaviours (diet, exercise, and medication management). Developed by the research team, the dynamic SOC charts for each of the modifiable health behaviours influencing the management of hypertension: medication management, diet, and exercise (see Additional file 1) consisted of a 5-item measure that was designed to assign participants to the stage into which they best fitted; the pre-contemplation, contemplation, preparation, action, or maintenance stage. The wording for each of the stages described a participant’s verbal cues at each stage and was based on a study that developed a similar scale for self-efficacy and the stages of exercise behaviour change [8, 25]. For this study, the wording was adapted for diet and medication management. Unlike previous SOC scales found within the literature, this tool displays the wording for each stage of change within a segment of a wheel, presenting the stages of change as dynamic and allowing for progression and regression. Data analysis was enabled by denoting a number according to a Likert scale: 1 = pre-contemplation; 2 = contemplation; 3 = preparation; 4 = action and 5 = maintenance. The stages of change charts for each of the modifiable health behaviours were completed at session one and session four by all study participants.

### Other measures

The secondary outcome measure was a change in systolic blood pressure (SBP) from session one after three (monthly) health coaching sessions provided by the community pharmacist. Blood pressure was measured using an automated blood pressure monitor (OMRON HEM-7121). Pharmacist health coach training included guidelines on blood pressure assessments. The guidelines stated that the participant should be seated with their feet flat on the floor, legs uncrossed, upper arm bare and with their back and arm supported. Two recordings were taken 1 minute apart, and the lower of the two recordings was recorded.

To support the outcomes of the dynamic SOC charts, health coaching participants also completed the Adherence to Refills and Medications Scale (ARMS) questionnaire, a validated self-report adherence scale [26]. The ARMS scale contains twelve questions to assess a participant’s medication adherence, which are divided into two categories, adherence with taking medications (eight items) and adherence with refilling prescriptions (four items). Each question is scored on a 4-point scale: 1 = none; 2 = some; 3 = most; 4 = all. Possible scores range from 12 to 48, with a lower score indicating greater adherence. The twelve-item scale has high internal consistency overall (Cronbach’s alpha = 0.80) [27]. The internal consistency of ARMS in this study was calculated to be 0.74.

### Data analysis

Descriptive statistics were used to summarise the behaviour change scores for intervention and control group participants. All statistical analyses were performed using the software program IBM SPSS-23 with the significance level set at *p* < 0.05. A test for normality showed that some of the study data were not normally distributed and therefore non-parametric tests were used where required. The Mann-Whitney *U* test was used to compare and validate the behaviour change scores at session one and session four between the participants that received pharmacist health coaching and the participants who did not. The Wilcoxon Signed-Rank test was used to compare the difference in systolic blood pressure from session one and session four. The effect size (*d*) was calculated for each outcome to quantify the difference between the extent to which health coaching influenced changes to the modifiable health behaviours and systolic blood pressure reduction. An effect size is classified as small (*d* = 0.2), medium (*d* = 0.5) and large (*d* > 0.8) [28]. Paired-sample *t*-tests were used to assess the outcomes of the ARMS questionnaire, from session one to session four.

## Results

### Pharmacists

Of the five pharmacies approached for the study, four did not recruit any participants for the study and thus were excluded from the trial. The trial was subsequently conducted at one community pharmacy. Two pharmacists provided verbal and written information about the study to eligible participants and requested that participants read and sign the informed consent document if they wished to take part in the study.

### Stages of change

A total of 20 participants met the inclusion criteria and received three pharmacist health coaching sessions at the community pharmacy at monthly intervals. The results of the Wilcoxon Signed-Rank test for within-group comparison are shown in Table 1. Participants who received health coaching experienced statistically significant changes from session one to session four in medication management from a mean of 4.19 to 4.65 (*p* = 0.03, *d* = 0.19) and exercise-related behaviour change from a mean of 3.05 to 4.05 *(p* = 0.01, *d* = 0.85). Participants diet-related health behaviours changed from a mean of 3.95 to 4.55 (*p* = 0.08, d = 0.63), which was not statistically significant.

**Table 1:** Comparison of behaviour change scores between the control group and health coaching group and a comparison of Adherence to Refills and Medications Scale (ARMS) scores and blood pressure results in the health coaching group

Behaviour Change		Session 1	Session 4	***Z***	***p***	***d***
*Medication management*						
***Control group***	*M (SD)* ***±*** *CI*	4.20(1.23) ± 0.54	4.20 (0.23) ±2.04	0.00	1.00^c^	0.00^c^
***Health coaching group***	*M (SD)* ***±*** *CI*	4.19 (0.83) ±1.84	4.65 (0.49) ±0.21	2.12	0.03^c^ 0.68^a^ 0.42^b^	0.19^c^ −0.10^a^ 1.25^b^
*Exercise*						
***Control group***	*M (SD)* ***±*** *CI*	3.30 (1.23) ±0.54	3.60 (1.07) ±0.47	1.34	0.18^c^	0.26^c^
***Health coaching group***	*M (SD)* ***±*** *CI*	3.05 (1.40) ±0.61	4.05 (0.94) ±0.41	2.63	0.01^c^ 0.75^a^ 0.50^b^	0.85^c^ −1.19^a^ 0.45^b^
*Diet*						
***Control group***	*M (SD)* ***±*** *CI*	4.20(1.23) ±0.54	4.20(1.23) ±0.54	0.00	1.00^c^	0.00^c^
***Health coaching group***	*M (SD)* ***±*** *CI*	3.95 (1.07) ±0.47	4.55 (0.82) ±0.36	1.77	0.08^c^ 0.48^a^ 0.01^b^	0.63^c^ −0.22^a^ 0.34^b^
*Blood Pressure*						
***Health coaching group***	*M (SD)* ***±*** *CI*	138.53 (15.41) ± 6.77	131.60 (17.10) ± 7.49	−2.10	.004	−0.42
*ARMS Score*						
***Health coaching group***	*M (SD) ± CI*	15.60 (3.38) ± 1.50	13.05 (1.50) ±0.66	3.64*	0.005	−1.05

^*a*^ *p-value is calculated by Mann- Whitney U test for between-groups comparison at session one*

^*b*^ *p-value is calculated by Mann- Whitney U test for between-groups comparison at session four*

^*c*^ *p-value is calculated by Wilcoxon Signed Rank test for within-group comparisons at session one and session four*

*t value

As hypothesised, no statistically significant difference in the SOC between session one and session four was apparent for the participants in the control group (Table 1). The results of the Mann- Whitney *U* test for between-group comparison (Table 1) indicates that there were no differences in the SOC between control group participants and those that received pharmacist health coaching at session one. In addition, there were no differences in the SOC between groups at session four for medication and, though some changes occurred to participants’ diet, where mean SOC scores changed from 4.20 to 4.55 (*p* = 0.01, *d =* 0.34).

### Blood pressure

**Fig. 1:**
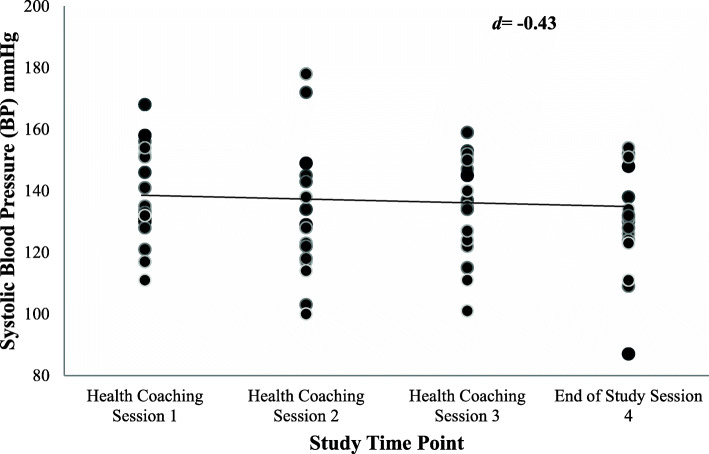
Systolic blood pressures for all patients at four-time points in the study showing a line of best fit

Systolic blood pressure was measured at each health coaching session. Although there was variability between the individual participants’ blood pressures at each time point (Fig. 1), the Wilcoxon Signed-Rank test showed that an overall reduction in mean systolic blood pressure was apparent at session four. The mean systolic blood pressure improved significantly from 138.53 mmHg at session one to 131.60 mmHg at session four, with an effect size of − 0.42 (*p* < 0.05) (Fig. 1 and Table 1). Given that the participants had poorly controlled hypertension for some participants blood pressure readings recorded at the enrolment session were higher than at session 1.

### Adherence to refills and medications (ARMS)

The paired-sample *t*-test showed that the mean scores for ARMS decreased significantly from 15.60 at session one to a mean of 13.05 at session four (*p* < 0.05) (Table 1). The reduction in the mean ARMS score suggests that the pharmacist health coaching intervention improved adherence to antihypertensive medications.

## Discussion

Previously, the application of the SOC model has involved the use of interviews and questionnaires. Although questionnaires are a convenient way for health professionals to collect information, they can often be cumbersome for participants [29]. For this reason, existing questionnaires used to assess SOC for diet, exercise, and medication adherence were adapted into dynamic SOC charts [8, 30, 31].

Although pharmacist health coaching studies at times have acknowledged the SOC theory, few have investigated its practical use [14, 16, 17]. The use of the TTM by pharmacists more broadly could encourage behaviour change in participants guiding participants to move forward in the SOC, as has been shown to improve smoking cessation related health behaviours [32]. In this study, the combined approach included education about clinical targets, lifestyle changes, and medication adherence specific to the participant’s needs and values. It was tailored to the participants’ SOC to encourage progress towards positive behaviour changes for diet, exercise, medication management, and adherence; however, since the movement is dynamic some participants also regressed to earlier stages [33]. Although the results from the current study appear consistent with prior research, the method introduced in the current study has the advantage of incorporating both dialogue, SOC charts and health coaching thus providing the pharmacist with a comprehensive visual representation of the participant’s initial health status and progress at subsequent health coaching sessions. It also enables the pharmacist to tailor their coaching strategies accordingly by providing ‘stage-directed counselling’. Stage directed counselling involves harnessing and encouraging the skills and motivation that participants’ already possess at their current stage to help them to continue to move in a positive direction through the SOC. [34] In comparison, a more direct style of counselling such as generic pharmacist counselling does not enable recognition of a participant’s SOC. It involves a linear, one-way process lacking participant-centred discussions [35], and is unlikely to facilitate progress from pre-contemplation unless the participant is already in the later stages of change.

Potentially, given that the health coaching group participants were intrinsically motivated to participate in the trial; greater dissatisfaction would have been experienced when changes to the clinical parameters measuring their modifiable health behaviours, medication management, exercise, and diet, were not evident at coaching session two. Consequently, some of these participants cycled back towards an earlier stage of change. This dynamic movement is also apparent in Fig. 1, with some health coaching participants experiencing moderate increases in blood pressure at health coaching session two in comparison to session three. However, at the conclusion of the study, it was apparent the intervention group participants experienced an overall positive movement through the SOC. This outcome supports the literature stating that extended participant support through health coaching leads to motivation and prevents a participant from returning to their previous unhealthy routine. For those that suffer from chronic disease, these behaviour changes are imperative to long term disease prognosis [36].

The results of the Mann Whitney *U* test for between-groups comparison indicated that there were no differences at session one (Table 1). This outcome is favourable for the purpose of control assessments [37] as it demonstrates that participants in both groups were at similar SOC for the three modifiable health behaviours- medication management, exercise and diet at session one. The results of the Mann Whitney *U* test results for between-group differences at session four showed that no changes were evident for the modifiable health behaviours medication management and exercise at, though a significant difference was apparent for the modifiable health behaviour diet (*p* = 0.01, *d* = 0.34). These differences may be attributable to the small number of control participants; larger cohort studies are necessary to confirm these findings.

Despite the variability in the individual participants’ systolic blood pressure at the four study time points (Fig. 1), a statistically significant change was apparent from session one to session four. It is important to note that all participants reported poorly controlled blood pressure before commencing the coaching trial. The degree of variability is typical for a sample that is ecologically valid in that it captures what pharmacists are usually faced with in terms of patient variability. The results of the current study also support the finding from previous pharmacist health coaching studies where participants with hypertension showed improvements to blood pressure [16], adherence to antihypertensive medications [15, 17], and confidence in self-management of their condition [14].

The change in participants’ systolic hypertension coincided with the decrease in ARMS scores from session one to session four, with a lower score indicating greater medication adherence. The proportion of participants who experienced a positive shift within the stages of change for the modifiable health behaviour medication management also correlates with this distribution. The changes to ARMS scores demonstrate that although most participants were initially adherent to their medications at session one, they further improved throughout the health coaching study.

### Study limitations

Unlike previous methods used to assess SOC, dynamic charts have been used in this study to capture the cyclic process of behaviour change. This produces more useful data interpretation of the intervention. However, as with the conventional methods used to assess SOC, the dynamic method also has limitations. It is plausible that participant biases could have skewed some of the behaviour change data, as participants may have been unwilling to report accurately on their SOC for the health behaviours, medication management, exercise, and diet. Biases may also be associated with participants misinterpreting the statements within the behaviour change charts. Furthermore, it must also be recognised that within-group analysis can lead to false-positive results and therefore it may not be appropriate to make comparisons within groups [38].

Given the small sample size of this research, and that the control group was not assessed for changes to clinical outcomes for the study period, other limitations must be acknowledged. It may not be appropriate to generalise the results to the wider population and the sustainability of the changes. Another limitation to the present study is that only a single site agreed to and recruited participants for the study. In addition, there may have been variability in demographic characteristics between the groups, which could have influenced the results of the between-group comparisons. Therefore, the results cannot be generalised to the outcomes that could be experienced by participants from another pharmacy and location. Thus, we suggest that larger control group studies be conducted to support the findings.

## Conclusion

This study supports the training of Australian community pharmacists to provide health coaching to improve the management of chronic health conditions such as hypertension. The study also demonstrated that trained pharmacists could apply the stages of change theory to assist a participant’s management of chronic health conditions by focusing on modifiable health behaviours. The use of the dynamic stages of change charts may allow pharmacists trained in behaviour change techniques to recognise a participant’s readiness to change health behaviours visually. The data obtained from the charts are associated with changes to systolic blood pressure, and medication adherence, and thus could be an effective tool to guide health interventions.

### Practical implication

This study provides evidence to support the training of Australian community pharmacists to health coach and adopt the TTM. Together, they allow participant-centred stage-directed health coaching to begin promptly, facilitating immediate action, and progress through the SOC. The results bring forward a means of improved participant care and health outcomes in particular for those with chronic health conditions.

## Supplementary Information

**Additional file 1:** Stages of change chart- Medication management. Stages of change chart- Exercise. Stages of change chart- Diet

**Additional file 2.**

## Data Availability

The study data and material have been securely stored electronically and can be requested by contacting the corresponding author.

## Abbreviations

TTM: Transtheoretical model
BP: Blood pressure
GP: General practitioner
SOC: Stages of change
US: The United States of America
