# Supplementary material for: A pharmacist health coaching trial evaluating behavioural changes in participants with poorly controlled hypertension
Source: BMC Fam Pract. 2021 Feb 14;22:35. doi: 10.1186/s12875-021-01385-0 (PMC7883432; doi:10.1186/s12875-021-01385-0)
Supplement: Supplementary file 1 — Additional file 1: Stages of change chart- Medication management. Stages of change chart- Exercise. Stages of change chart- Diet [file 12875_2021_1385_MOESM1_ESM.docx]

**Appendix 1**

**Stages of change chart- Medication management Stages of change chart- Exercise**

**1.**

“I don’t think I need to take my blood pressure medication.”

**2.**

“I know that it is important to take my blood pressure medications, but I forget to take them.”

**3.**

“I know that I need to take my blood pressure medications and I will try to remember to take it.”

**4.**

“I have a routine with my blood pressure medication in order to remind me to take it every day.”

**5.**

“I have been taking my blood pressure medications everyday and I don’t forget

**1.**

“I’m not really interested in exercising. I don’t believe I need to.”

**2.**

“I know I need to exercise, but with all that going on in my life right now, I’m not too sure if I can.”

**3.**

“I have to exercise, and I’m planning to do that.”

**4.**

“I’m doing my best to exercise regularly.”

**5.**

“I’ve been exercising consistently and will keep it up

**Stages of change chart- Diet**

**1.**

“I’m not really interested in changing my diet. My diet is not a problem.”

**2.**

“I know my diet isn’t too good, but with all that’s going on in my life right now, I’m not sure I can.”

**3.**

“I have to change my diet, and I am planning on doing that.”

**4.**

“I’m doing my best to change my diet; this is harder than I thought.”

**5.**

“I’ve been eating well and I have seen improvements in my health. I will keep this up.”
