# Supplementary material for: A pharmacist health coaching trial evaluating behavioural changes in participants with poorly controlled hypertension
Source: BMC Fam Pract. 2021 Feb 14;22:35. doi: 10.1186/s12875-021-01385-0 (PMC7883432; doi:10.1186/s12875-021-01385-0)
Supplement: Supplementary file 2 — Additional file 2. [file 12875_2021_1385_MOESM2_ESM.docx]

**Visit 1**

**SCREENING**

**Date: ____ /____ /____**

1. **Participant Initials:** _________________________
2. **Date of Birth:**_____________________
3. **Age:**____________________________
4. **Would you like us to inform your doctor about your progress throughout the study?**

No ⬜

Yes ⬜ (If yes) Doctor Contact Details

Name: ______________________________________________

Address: ____________________________________________

____________________________________________________

1. **Current Medications**

| **Medication** | **Used for** |
| --- | --- |
|  |  |
|  |  |
|  |  |
|  |  |
|  |  |

1. **Inclusion Criteria**

All items must be marked ‘Yes’ for the participant to be eligible.

|  | **No** | **Yes** |
| --- | --- | --- |
| Aged >18 years | ⬜ | ⬜ |
| Systolic and/or diastolic hypertension (≥ 140/90 mmHg) despite compliance with at least one antihypertensive | ⬜ | ⬜ |
| Recognised as having uncontrolled hypertension by the pharmacist (in the form of participant/pharmacy records for a period of 3 months) | ⬜ | ⬜ |
| Understands English language | ⬜ | ⬜ |
| Non/ Ex-smoker | ⬜ | ⬜ |

1. **Exclusion criteria**

All items must be marked ‘No’ for the participant to be eligible.

|  | **No** | **Yes** |
| --- | --- | --- |
| Aged ≤18 years | ⬜ | ⬜ |
| Has experiences Myocardial Infarction, stroke, angina attack within the previous 6 months | ⬜ | ⬜ |
| Is pregnant | ⬜ | ⬜ |
| Current Smoker | ⬜ | ⬜ |
| Secondary hypertension, cardiovascular (CVD) disorders (unstable angina pectoris, heart failure, life-threatening arrhythmia, nephropathy, and grade III-IV retinopathy), intolerance to ambulatory blood pressure (BP) monitoring (ABPM) | ⬜ | ⬜ |
| Inability to communicate and comply with all study requirements | ⬜ | ⬜ |
| Requires diuretics, CCB, ACEI or α-blockers for reasons other than hypertension | ⬜ | ⬜ |
| Has work/life commitments that may interfere with study requirements | ⬜ | ⬜ |

1. **Does the participant meet the eligibility criteria** YES ⬜ NO ⬜ (exclude from study)
2. **Has the participant signed the participant informed consent forms (PICF)?** YES ⬜ No ⬜

**Visit 2**

**BASELINE ASSESSMENTS**

**Date: ____ /____ /____**

1. **Number code assigned___________________**
2. **Gender:**  FEMALE ⬜ MALE ⬜
3. **Allergies:**____________________________________________________________________
4. **Ethnicity______________________________________________**
5. **Occupation___________________________________________**
6. **Blood Pressure: [Participants should be relaxed and seated for five minutes prior to readings]**
   1. **Reading #1:** Systolic ____________ mmHg Diastolic___________ mmHg

***Wait 1 minute***

- 1. **Reading #2** Systolic ____________ mmHg Diastolic___________ mmHg

**Lowest reading**  Systolic ____________ mmHg Diastolic___________ mmHg

1. **Height:** _______________ cm
2. **Weight:** ______________ kg
3. **BMI= weight (kg)/ height^2^ (m^2^)** _________________
4. **Waist circumference:**_________________ cm

**Advise participant that now you would like to turn on a the audio recorder. Please inform participants that they have agreed to have these sessions audio recorded and that they can switch off the recording at any time. Also advise participants that you may also take notes during this time.**

1. **Current Medications to control blood pressure:**

|  |  |
| --- | --- |
|  |  |
|  |  |

1. **a. What do you use each of the medications for?**

**b. How do they work?**

| **1a** | **b** |
| --- | --- |
| **2a** | **b** |
| **3a** | **b** |
| **4a** | **b** |
| **5a** | **b** |
| **6a** | **b** |

1. **Some people often forget to take their medicines. How do you remember to take yours?**
   1. *Please explain the role of each antihypertensive medication to the participant.*
2. **Provide medication adherence questionnaire [advise participants that there are no right or wrong answer]**

**Adherence to refills and medication scale (ARMS)**

| Survey Items | None | Some | Most | All |
| --- | --- | --- | --- | --- |
| 1. How often do you forget to take you medicine? | 1 | 2 | 3 | 4 |
| 1. How often do you decide not to take your medicine? | 1 | 2 | 3 | 4 |
| 1. How often do you forget to get prescriptions filled? | 1 | 2 | 3 | 4 |
| 1. How often do you run out of medicine? | 1 | 2 | 3 | 4 |
| 1. How often do you skip a dose of your medicine before you go to the doctor? | 1 | 2 | 3 | 4 |
| 1. How often do you miss taking your medicine when you feel better? | 1 | 2 | 3 | 4 |
| 1. How often do you miss taking your medicine when you feel sick? | 1 | 2 | 3 | 4 |
| 1. How often do you miss taking your medicine when you are careless? | 1 | 2 | 3 | 4 |
| 1. How often do you miss taking your medicine? | 1 | 2 | 3 | 4 |
| 1. How often do you change the dose of your medicine to suit your needs (like when you take more or less pills than you are supposed to)? | 1 | 2 | 3 | 4 |
| 1. How often do you put off refilling you medicines because they cost too much? | 1 | 2 | 3 | 4 |
| 1. How often do you plan ahead and refill your medicines before they run out? | 1 | 2 | 3 | 4 |

**Visit 3**

**HEALTH COACHING SESSION 1**

**Date: ____ /____ /____**

Introduction/ Greeting ⬜

**Advise participant that now you would like to turn on the audio recorder. Please inform participants that they have agreed to have these sessions audio recorded and that they can switch off the recording at any time. Also advise participants that you may also take notes during this time.**

Questions to ask the participant:

1. Where do you think you are in terms of your blood pressure management?
   1. Refer to stages of change charts (exercise diet and medication management) and ask participant to mark where they lie (see next page) ⬜
2. What do you know about health coaching? ⬜
   1. *Inform the participant about health coaching*  ⬜
3. What do you know about high blood pressure? ⬜
4. How do you get high blood pressure? ⬜
5. What does it do to your body? ⬜
6. What is the target blood pressure for someone that has hypertension? ⬜
7. *Educate participant about hypertension and provide pharmacy care card on*

*high blood pressure to participant* ⬜

1. What is your blood pressure? ⬜
2. What would you like your blood pressure to be? ⬜
3. How does you high blood pressure affect your life? ⬜
4. How do you manage your high blood pressure? ⬜
5. What could you do to improve this over the next month? ⬜
6. *Write down goals and plan on the goal setting worksheet for this visit* ⬜
7. Summarise the participants goals and plan of attainment ⬜
8. *Provide participant with summary (photocopy worksheet for this visit)* ⬜
9. Is there anything else that you would like to talk about today? ⬜
10. Advise participant what to do if they experience any signs of an adverse event: ⬜
11. ***Contact the pharmacy or their doctor if they have any concerns about their health and to call triple 000 in cases of emergency.*** ⬜

**Visit 3**

**Stages of change chart- Medication management**

**Please mark on the chart the description that best describes you**

Pharmacist comments:

__________________________________________________________________________________________________________________________________________________________________________________________________________________________________________________________________________________________________________________________________________________________________________________________________________________________________________________________________________________________________________________________________________________________________________________________________________________________________________________________________

**3.**

“I have been taking my blood pressure medications everyday and I don’t forget

**4.**

“I have a routine with my blood pressure medication in order to remind me to take it every day.”

**5.**

“I know that I need to take my blood pressure medications and I will try to remember to take it.”

**2.**

“I know that it is important to take my blood pressure medications, but I forget to take them.”

**1.**

“I don’t think I need to take my blood pressure medication.”

**Visit 3**

**Stages of change chart- Exercise**

**Please mark on the chart the description that best describes you**

Pharmacist comments:

__________________________________________________________________________________________________________________________________________________________________________________________________________________________________________________________________________________________________________________________________________________________________________________________________________________________________________________________________________________________________________________________________________________________________________________________________________________________________________________________________

**5.**

“I’ve been exercising consistently and will keep it up

**4.**

“I’m doing my best to exercise regularly.”

**3.**

“I have to exercise, and I’m planning to do that.”

**2.**

“I know I need to exercise, but with all that going on in my life right now, I’m not too sure if I can.”

**1.**

“I’m not really interested in exercising. I don’t believe I need to.”

**Visit 3**

**Stages of change chart- Diet**

**Please mark on the chart the description that best describes you**

Pharmacist comments:

__________________________________________________________________________________________________________________________________________________________________________________________________________________________________________________________________________________________________________________________________________________________________________________________________________________________________________________________________________________________________________________________________________________________________________________________________________________________________________________________________

**5.**

“I’ve been eating well and I have seen improvements in my health. I will keep this up.”

**4.**

“I’m doing my best to change my diet; this is harder than I thought.”

**3.**

“I have to change my diet, and I am planning on doing that.”

**2.**

“I know my diet isn’t too good, but with all that’s going on in my life right now, I’m not sure I can.”

**1.**

“I’m not really interested in changing my diet. My diet is not a problem.”

**Stages of change charts**

**Pharmacists please comment in the sections provided on each cart:**

- **Which stage of do you believe the participant is at?**

**1. Pre-contemplation, 2. Contemplation, 3. Preparation, 4. Action, 5. Maintenance**

- **Why do you believe the participant is at this stage? (please explain)**

**Goal Setting Performa for Visit 3**

*Please provide a copy of this to the participant*

| **My blood pressure last month was:**  **__________ /__________mmHg** | **My current blood pressure is:**  **__________ /__________mmHg** | **My personal blood pressure goal for this month is:**  **__________ /__________mmHg** |
| --- | --- | --- |
| **My personal action plan for this month**  I will work towards improving my blood pressure by changing my:   1. Medication management   ____________________________________________________________________________________________________________________________________________________________________________________________________________________________________________________________________________________________________________   1. Exercise   ____________________________________________________________________________________________________________________________________________________________________________________________________________________________________________________________________________________________________________   1. Medication Management   ____________________________________________________________________________________________________________________________________________________________________________________________________________________________________________________________________________________________________________ | | |

**Visit 4**

**HEALTH COACHING SESSION 2**

**Date: ____ /____ /____**

Introduction/ Greeting ⬜

**Advise participant that now you would like to turn on the audio recorder. Please inform participants that they have agreed to have these sessions audio recorded and that they can switch off the recording at any time. Also advise participants that you may also take notes during this time.**

Questions to ask the participant:

1. Have you experiences and adverse events (AE’s)? ***Record on next page***  ⬜
2. Can I check your blood pressure (BP)? ***Record on next page***  ⬜
   1. Check and record the BP it in the participants notes and participants goal setting worksheet ⬜
   2. Compare this BP reading with the previous month and discuss with participant ⬜
3. What was the goal you set for yourself at our last session?
   1. Remind if necessary ⬜
4. Did you reach your goal?

| Yes ⬜ | No ⬜ |
| --- | --- |
| You should be proud of the hard work that you have put into achieving your goals  🡫  Do you think that the goals you set for yourself were too ambitious, too cautious or just right?  🡫  When you think about the goals you set yourself, how does it make you feel?  🡫  If you were to set new goals for the next month what would it be? | Looks like you tried very hard and were close to achieving your goals  🡫  Do you think that the goals you set for yourself were too ambitious, too cautious or just right?  🡫  When you think about the goals you set yourself, how does it make you feel?  🡫  If you were to set new goals for the next month what would it be? |

1. How will you reach these goals?
2. Write down goals and plan on the goal setting worksheet for this visit ⬜
3. Summarise the participants goals and plan of attainment ⬜
4. Provide participant with summary (photocopy worksheet for this visit) ⬜
5. Is there anything else that you would like to talk about today? ⬜
6. Advise participant what to do if they experience any signs of an adverse event: ⬜
7. ***Contact the pharmacy or doctor if they have any concerns about their health and to call triple 000 in cases of emergency***.

**ADVERSE EVENT (Visit 4)**

Date ___/___/___

Did any adverse event occur since the last visit? YES⬜ NO⬜

If yes, please explain the adverse event

___________________________________________________________________________________________________________________________________________________________________________________________________________________________________________________

**BLOOD PRESSURE (Visit 4)**

**Blood Pressure: [Participants should be relaxed and seated for five minutes prior to readings]**

- 1. **Reading #1:** Systolic ____________ mmHg Diastolic___________ mmHg

***Wait 1 minute***

- 1. **Reading #2** Systolic ____________ mmHg Diastolic___________ mmHg

**Lowest reading**  Systolic ____________ mmHg Diastolic___________ mmHg

**Goal Setting Performa for Visit 4**

*Please provide a copy of this for the participant*

| **My blood pressure last month was:**  **________ /________mmHg** | **My current blood pressure is:**  **________ /________mmHg** | **My personal blood pressure goal for this month is:**  **________ /________mmHg** |
| --- | --- | --- |
| **My personal action plan for this month**  I will work towards improving my blood pressure by changing my:   1. Medication management   ____________________________________________________________________________________________________________________________________________________________________________________________________________________________________________________________________________________________________________   1. Exercise   ____________________________________________________________________________________________________________________________________________________________________________________________________________________________________________________________________________________________________________   1. Medication Management   ____________________________________________________________________________________________________________________________________________________________________________________________________________________________________________________________________________________________________________ | | |

**Visit 5**

**HEALTH COACHING SESSION 3**

**Date: ____ / ____ /____**

Introduction/ Greeting ⬜

**Advise participant that now you would like to turn on the audio recorder. Please inform participants that they have agreed to have these sessions audio recorded and that they can switch off the recording at any time. Also advise participants that you may also take notes during this time.**

Questions to ask the participant:

1. Have you experiences and adverse events (AE’s)? ***Record on next page***  ⬜
2. Can I check your blood pressure (BP)? ***Record on next page***  ⬜
   1. Check and record the BP it in the participants notes and participants goal setting worksheet ⬜
   2. Compare this BP reading with the previous month and discuss with participant ⬜
3. What was the goal you set for yourself at our last session?
   1. Remind if necessary ⬜
4. Did you reach your goal?

| Yes ⬜ | No ⬜ |
| --- | --- |
| You should be proud of the hard work that you have put into achieving your goals  🡫  Do you think that the goals you set for yourself were too ambitious, too cautious or just right?  🡫  When you think about the goals you set yourself, how does it make you feel?  🡫  If you were to set new goals for the next month what would it be? | Looks like you tried very hard and were close to achieving your goals  🡫  Do you think that the goals you set for yourself were too ambitious, too cautious or just right?  🡫  When you think about the goals you set yourself, how does it make you feel?  🡫  If you were to set a new goals for the next month what would it be? |

1. How will you reach these goals?
2. Write down goals and plan on the goal setting worksheet for this visit ⬜
3. Summarise the participants goals and plan of attainment ⬜
4. Provide participant with summary (photocopy worksheet for this visit) ⬜
5. Is there anything else that you would like to talk about today? ⬜
6. Advise participant what to do if they experience any signs of an adverse event: ⬜
7. ***Contact the pharmacy or doctor if they have any concerns about their health and to call triple 000 in cases of emergency***.

**ADVERSE EVENT (Visit 5)**

Date ___/___/___

Did any adverse event occur since the last visit? YES⬜ NO⬜

If yes, please explain the adverse event

___________________________________________________________________________________________________________________________________________________________________________________________________________________________________________________

**BLOOD PRESSURE (Visit 5)**

**Blood Pressure: [Participants should be relaxed and seated for five minutes prior to readings]**

- 1. **Reading #1:** Systolic ____________ mmHg Diastolic___________ mmHg

***Wait 1 minute***

- 1. **Reading #2** Systolic ____________ mmHg Diastolic___________ mmHg

**Lowest reading**  Systolic ____________ mmHg Diastolic___________ mmHg

**Goal Setting Performa for Visit 5**

*Please provide a copy of this for the participant*

| **My blood pressure last month was:**  **________ /________mmHg** | **My current blood pressure is:**  **________ /________mmHg** | **My personal blood pressure goal for this month is:**  **________ /________mmHg** |
| --- | --- | --- |
| **My personal action plan for this month**  I will work towards improving my blood pressure by changing my:   1. Medication management   ____________________________________________________________________________________________________________________________________________________________________________________________________________________________________________________________________________________________________________   1. Exercise   ____________________________________________________________________________________________________________________________________________________________________________________________________________________________________________________________________________________________________________   1. Medication Management   ____________________________________________________________________________________________________________________________________________________________________________________________________________________________________________________________________________________________________________ | | |

**Visit 6**

**END OF STUDY**

**Date: ____ / ____ /____**

Introduction/ Greeting ⬜

**Advise participant that now you would like to turn on the audio recorder. Please inform participants that they have agreed to have these sessions audio recorded and that they can switch off the recording at any time. Also advise participants that you may also take notes during this time.**

Questions to ask the participant:

1. Have you experiences and adverse events (AE’s)? ***Record on next page***  ⬜
2. Can I check your blood pressure (BP)? ***Record on next page***  ⬜
   1. Check and record the BP it in the participants notes and participants goal setting worksheet ⬜
   2. Compare this BP reading with the previous month and discuss with participant ⬜
3. Can I check your weight and waist circumference? ⬜
4. Compare to beginning of study ⬜
5. What was the goal you set for yourself at our last session? ⬜
6. Remind if necessary ⬜
7. Did you reach your goal? ⬜

| Yes ⬜ | No ⬜ |
| --- | --- |
| You should be proud of the hard work that you have put into achieving your goals  🡫  What challenges did you face along the way?  🡫  I hope that the skills you have learnt will help you keep track of your BP | Looks like you tried very hard and were close to achieving your goal  🡫  What challenges did you face along the way?  🡫  Keep it up!  🡫  I hope that the skills you have learnt will help you keep track of your BP |

1. Ask the participant to determine which stage of change they are at from the stages of change chart s ***(see following pages)***
2. Provide medication adherence questionnaire [advise participants that there are no right or wrong answer ***(see following page)***
3. Before we finish off this study, I would like to ask you some of the questions I asked you at the beginning of the study, it that okay?
   1. What do you know about hypertension? ⬜
   2. How do you get hypertension? ⬜
   3. What does hypertension do to your body? ⬜
   4. What is the target blood pressure for someone that has hypertension? ⬜
   5. How do you manage your hypertension? ⬜
   6. How did you feel about the health coaching sessions? ⬜
   7. Do you think that this is a service that pharmacies should offer? ⬜
   8. Is there anything else that you would like to talk about? ⬜

Parting phrase

-End of Study-

**ADVERSE EVENT (Visit 6)**

Date ___/___/___

Did any adverse event occur since the last visit? YES⬜ NO⬜

If yes, please explain the adverse event

___________________________________________________________________________________________________________________________________________________________________________________________________________________________________________________

**BLOOD PRESSURE (Visit 6)**

**Blood Pressure: [Participants should be relaxed and seated for five minutes prior to readings]**

1. **Reading #1:** Systolic ____________ mmHg Diastolic___________ mmHg

***Wait 1 minute***

1. **Reading #2** Systolic ____________ mmHg Diastolic___________ mmHg

**Lowest reading**  Systolic ____________ mmHg Diastolic___________ mmHg

**Visit 6**

**Stages of change chart- Medication management**

**Please mark on the chart the description that best describes you**

**3.**

“I have been taking my blood pressure medications everyday and I don’t forget

**4.**

“I have a routine with my blood pressure medication in order to remind me to take it every day.”

**5.**

“I know that I need to take my blood pressure medications and I will try to remember to take it.”

**2.**

“I know that it is important to take my blood pressure medications, but I forget to take them.”

**1.**

“I don’t think I need to take my blood pressure medication.”

Pharmacist comments:

__________________________________________________________________________________________________________________________________________________________________________________________________________________________________________________________________________________________________________________________________________________________________________________________________________________________________________________________________________________________________________________________________________________________________________________________________________________________________________________________________

Pharmacist comments:

__________________________________________________________________________________________________________________________________________________________________________________________________________________________________________________________________________________________________________________________________________________________________________________________________________________________________________________________________________________________________________________________________________________________________________________________________________________________________________________________________

**5.**

“I’ve been exercising consistently and will keep it up

**4.**

“I’m doing my best to exercise regularly.”

**3.**

“I have to exercise, and I’m planning to do that.”

**2.**

“I know I need to exercise, but with all that going on in my life right now, I’m not too sure if I can.”

**1.**

“I’m not really interested in exercising. I don’t believe I need to.”

**Visit 6**

**Stages of change chart- Exercise**

**Please mark on the chart the description that best describes you**

**5.**

“I’ve been eating well and I have seen improvements in my health. I will keep this up.”

**4.**

“I’m doing my best to change my diet; this is harder than I thought.”

**3.**

“I have to change my diet, and I am planning on doing that.”

**2.**

“I know my diet isn’t too good, but with all that’s going on in my life right now, I’m not sure I can.”

**1.**

“I’m not really interested in changing my diet. My diet is not a problem.”

Pharmacist comments:

__________________________________________________________________________________________________________________________________________________________________________________________________________________________________________________________________________________________________________________________________________________________________________________________________________________________________________________________________________________________________________________________________________________________________________________________________________________________________________________________________

Pharmacist comments:

__________________________________________________________________________________________________________________________________________________________________________________________________________________________________________________________________________________________________________________________________________________________________________________________________________________________________________________________________________________________________________________________________________________________________________________________________________________________________________________________________

**1.**

“I’m not really interested in changing my diet. My diet is not a problem.”

**2.**

“I know my diet isn’t too good, but with all that’s going on in my life right now, I’m not sure I can.”

**3.**

“I have to change my diet, and I am planning on doing that.”

**4.**

“I’m doing my best to change my diet; this is harder than I thought.”

**5.**

“I’ve been eating well and I have seen improvements in my health. I will keep this up.”

**Visit 6**

**Stages of change chart- Diet**

**Please mark on the chart the description that best describes you**

**Stages of change charts**

**Pharmacists please comment in the sections provided on each cart:**

- **Which stage of do you believe the participant is at?**

**1. Pre-contemplation, 2. Contemplation, 3. Preparation, 4. Action, 5. Maintenance**

- **Why do you believe the participant is at this stage? (please explain)**

**Adherence to refills and medications scale (ARMS)**

| Survey Items | None | Some | Most | All |
| --- | --- | --- | --- | --- |
| 1. How often do you forget to take you medicine? | 1 | 2 | 3 | 4 |
| 1. How often do you decide not to take your medicine? | 1 | 2 | 3 | 4 |
| 1. How often do you forget to get prescriptions filled? | 1 | 2 | 3 | 4 |
| 1. How often do you run out of medicine? | 1 | 2 | 3 | 4 |
| 1. How often do you skip a dose of you medicine before you go to the doctor? | 1 | 2 | 3 | 4 |
| 1. How often do you miss taking your medicine when you feel better? | 1 | 2 | 3 | 4 |
| 1. How often do you miss taking your medicine when you feel sick? | 1 | 2 | 3 | 4 |
| 1. How often do you miss taking your medicine when you are careless? | 1 | 2 | 3 | 4 |
| 1. How often do you miss taking you medicine to | 1 | 2 | 3 | 4 |
| 1. How often do you change the dose of your medicine to suit your needs (like when you take more or less pills than you are supposed to)? | 1 | 2 | 3 | 4 |
| 1. How often do you put off refilling you medicines because they cost too much? | 1 | 2 | 3 | 4 |
| 1. How often do you plan ahead and refill your medicines before they run out? | 1 | 2 | 3 | 4 |

**Trial Participation Outcome**

Completed trial YES⬜ NO ⬜

Withdrawal from trial (complete withdrawal from below) YES⬜ NO ⬜

**Trial Withdrawal Form**

**Date of trial withdrawal ____ / ____ /____**

**Reason for withdrawal:**

Lost to follow up ⬜

Non-compliance ⬜

Concomitant medication ⬜

Medical contraindication ⬜

Adverse event (explain) ⬜

____________________________________________________________________________

Other (explain) ⬜

____________________________________________________________________________
